# Gonadotropin-Releasing Hormone Receptors in Prostate Cancer: Molecular Aspects and Biological Functions

**DOI:** 10.3390/ijms21249511

**Published:** 2020-12-14

**Authors:** Fabrizio Fontana, Monica Marzagalli, Marina Montagnani Marelli, Michela Raimondi, Roberta M. Moretti, Patrizia Limonta

**Affiliations:** Department of Pharmacological and Biomolecular Sciences, Università degli Studi di Milano, via Balzaretti 9, 20133 Milan, Italy; fabrizio.fontana@unimi.it (F.F.); monica.marzagalli@unimi.it (M.M.); marina.marellimontagnani@unimi.it (M.M.M.); michela.raimondi@unimi.it (M.R.); roberta.moretti@unimi.it (R.M.M.)

**Keywords:** castration-resistant prostate cancer (CRPC), GnRH receptors (GnRH-R), antiproliferative/proapoptotic activity, GnRH agonists, GnRH antagonists, cytotoxic GnRH-based bioconjugates

## Abstract

Pituitary Gonadotropin-Releasing Hormone receptors (GnRH-R) mediate the activity of the hypothalamic decapeptide GnRH, thus playing a key role in the regulation of the reproductive axis. Early-stage prostate cancer (PCa) is dependent on serum androgen levels, and androgen-deprivation therapy (ADT), based on GnRH agonists and antagonists, represents the standard therapeutic approach for PCa patients. Unfortunately, the tumor often progresses towards the more aggressive castration-resistant prostate cancer (CRPC) stage. GnRH receptors are also expressed in CRPC tissues, where their binding to both GnRH agonists and antagonists is associated with significant antiproliferative/proapoptotic, antimetastatic and antiangiogenic effects, mediated by the Gαi/cAMP signaling cascade. GnRH agonists and antagonists are now considered as an effective therapeutic strategy for CRPC patients with many clinical trials demonstrating that the combined use of these drugs with standard therapies (i.e., docetaxel, enzalutamide, abiraterone) significantly improves disease-free survival. In this context, GnRH-based bioconjugates (cytotoxic drugs covalently linked to a GnRH-based decapeptide) have been recently developed. The rationale of this treatment is that the GnRH peptide selectively binds to its receptors, delivering the cytotoxic drug to CRPC cells while sparing nontumor cells. Some of these compounds have already entered clinical trials.

## 1. Introduction

Gonadotropin-releasing hormone (GnRH) is the hypothalamic decapeptide (pGlu-His-Trp-Ser-Tyr-Gly-Leu-Arg-Pro-Gly-NH_2_) known to play a central role in the control of the hypothalamic-pituitary-axis in mammals [1,2,3,4]. It is produced by a small number of hypothalamic neurons and released in a pulsatile way into the hypophyseal circulation to reach the gonadotropes in the anterior pituitary where it binds to its specific receptors (GnRH-R). By binding to these receptors, GnRH triggers the synthesis and release of the two gonadotropoins LH (lutinizing hormone) and FSH (follicle stimulating hormone), thus stimulating gonadal sex steroid hormone production and gamete maturation in both sexes [5,6,7,8].

The pituitary GnRH-R is a protein (328 amino acids) belonging to the GPCR (rhodopsin-like G protein coupled receptor) family. It is encoded by a gene on chromosome 4q13.2 and consists of three exons interrupted by two introns (Table 1). The protein is structurally characterized by a core formed by seven transmembrane domains, an extracellular N-terminal domain (35 amino acids) and a typically short (1–2 amino acids) intracellular C-terminal domain [9,10,11,12]. This feature of GnRH-R has been linked to its slow internalization and desensitization upon hormone stimulation [13,14].

The intracellular signaling pathway triggered by GnRH-R activation at the pituitary level has been widely investigated and is now well characterized. Binding of the decapeptide to its receptors leads to the activation of the Gαq/11 subunit of a heterotrimeric G protein complex, stimulating, in turn, its direct effector phospholipase Cβ (PLCβ). PLCβ catalyzes the formation of diacylglycerol (DAG) and inositol triphosphate (IP_3_), leading to protein kinase C (PKC) activation and increased cytoplasmic levels of Ca^2+^ (due to increased ion influx from the extracellular environment and release from intracellular stores), respectively. Interestingly, different PKC isoforms were shown to be involved in these intracellular mechanisms. Activation of these PKC triggers their downstream signaling pathway involving proteins belonging to the MAPK (mitogen-activated protein kinase) cascade. In addition, elevated intracellular Ca^2+^ levels were also shown to be involved in the MAPK cascade activation [7,11,12,14,15,16,17,18,19,20,21]. In particular, Naor and coworkers recently reported that the PKC isoforms PKCα, PKCβII, PKCδ, PKCε, PKCθ and atypical PKC-ι/λ play differential roles in the ERK1/2, JNK1/2 and p38MAPK phosphorylation in a ligand and cell context-dependent manner. According to these authors, this may be related to “the persistent vs. transient redistribution of the different PKCs into the cell or the redistribution of a specific PKC from the perinuclear zone vs. the plasma membrane” [22,23]. GnRH-R-activated MAPKs then trigger the expression of gonadotropins as well as of GnRH-R genes.

This molecular cascade of events triggered by pituitary GnRH-R activation mediates the key regulatory role of GnRH in the control of the pituitary-gonadal axis functions.

It is now accepted that, in addition to the classical hypothalamic GnRH, other forms of the peptide are present in most vertebrates. In particular, the GnRH-II isoform is a decapeptide conserving the amino acids of GnRH in both the N-terminal (Glp-His-Trp-Ser) and the C-terminal (Pro-Gly-NH_2_) domains, suggesting that it might bind and activate the classical form of GnRH-R. On the other hand, the GnRH-II amino acid sequence differs from that of GnRH in positions 5, 7 and 8 (His^5^, Trp^7^, Tyr^8^), known to be involved in the biological functions of GnRH [4,12,24,25,26]. These observations stimulated the search for a specific receptor for GnRH-II (GnRH-II-R). However, so far, a full-length GnRH-II-R has been cloned in nonhuman primates [27,28]. In humans, Neill et al. proposed that the GnRH-II-R might be a five transmembrane domain receptor, lacking the transmembrane regions 1 and 2 [29]. Morgan and coworkers suggested the presence, in human tissues, of a nonfunctional form of this receptor, located on chromosome 1q12 and composed of three exons and two introns, as a consequence of a frameshift in exon 1 and a stop codon in exon 2 [30] (Table 1). Another splice variant of GnRH-II-R was reported in human sperm and suggested to have a functional role in male gametogenesis [31]. Thus, the existence of a functional seven transmembrane domain GnRH-II-R in human tissues is still a matter of debate.

A GnRH-III decapeptide was found in sea lamprey (*Petromyzon marinus*) with an amino acid sequence that differs from that of GnRH in positions 5–8 (His^5^, Asp^6^, Trp^7^, Lys^8^) [32]. This peptide was reported to be endowed with a very low LH and FSH-stimulating activity in rats [33].

It is now well established that GnRH-R are also expressed in extrapituitary tissues and in several tumor tissues, both related (prostate, breast, ovarian, endometrial tumors) and unrelated (melanoma, glioblastoma, pancreatic, colon, lung, adrenocortical head and neck tumors) to the reproductive system. In cancer cells, this receptor is associated with antiproliferative/proapoptotic effects [4,34,35,36,37,38,39,40,41,42,43,44,45,46].

Interestingly, the GnRH decapeptide was also shown to be expressed in these tumors, demonstrating the existence of an autocrine GnRH/GnRH receptor loop endowed with antitumor activity and supporting the role of this loop as an effective molecular target for anticancer strategies.

## 2. Prostate Cancer

Prostate cancer (PCa) still represents a major health burden, being the most aggressive tumor and the second most frequent cause of tumor-related deaths among men in western countries [47,48,49]. In the early stages, most PCas are dependent on androgens for their growth and, therefore, patients are treated with androgen-deprivation therapy (ADT, i.e., chemical castration). This therapeutic approach is mainly based on GnRH agonists, either alone or in combination with a drug targeting the androgen receptor signaling (antiandrogens, inhibitors of androgen synthesis).

GnRH agonists (goserelin, leuprorelin, triptorelin) were synthesized based on the observation that native GnRH is endowed with a short half-life, being enzymatically cleaved in the blood at the level of Gly^6^. Thus, this amino acid is replaced by a D-amino acid in order to obtain analogs resistant to the peptidase degradation. In these analogs, the first amino acids of GnRH are conserved to maintain the biological activity of the native peptide, while the last amino acid (Gly^10^-amide) is substituted with the residues Pro-NHEt or Pro-Azgly-NH_2_ to increase the binding affinity to the GnRH-R [50,51,52,53,54,55]. These compounds act by binding to the pituitary GnRH-R and, after the induction of an initial gonadotropin surge, they induce its desensitization, thus suppressing LH and testosterone secretion [56]. To avoid the risk of the initial flare event, together with metabolic dysfunction and increased risk of cardiovascular pathologies, GnRH antagonists were later synthesized (cetrorelix, abarelix, degarelix, ganirelix, ozarelix). They competitively bind to the GnRH-R immediately suppressing gonadotropin secretion. Moreover, they were shown to suppress FSH (follicle stimulating hormone) more rapidly, to lower levels and to a longer period than GnRH agonists [57,58]. These peptides present the Ac-D-Nal-D-Cpa-D-Pal sequence in their N-terminal domain, different D-amino acid derivatives in position 6 and D-Ala at their C-terminal domain [43,59,60,61]. Very recent data from clinical trials and meta-analyses support an improved progression-free survival, overall survival and side effects (cardiovascular diseases) with GnRH antagonists compared with GnRH agonists [55].

Given the key role of the androgen receptor (AR) signaling in PCa growth and progression, several agents targeting this molecular pathway were developed: (i) antiandrogens directly targeting the AR receptor such as bicalutamide, flutamide and nilutamide (first and second generation antiandrogens), and enzalutamide, apalutamide and darolutamide (third generation antiandrogens); (ii) inhibitors of intratumoral androgen synthesis such as finasteride, orteronel and abiraterone [43,62,63,64,65,66,67,68,69].

Unfortunately, the tumor often progresses towards the castration-resistant stage (castration-resistant prostate cancer, CRPC) characterized by uncontrolled progression in the absence of circulating androgens. Chemotherapy (docetaxel), either alone or in combination with antiandrogens (enzalutamide, apalutamide), or with inhibitors of androgen synthesis (abiraterone), represents the standard therapy for CRPC patients [68,70,71,72,73,74,75]. However, undesired side effects and development of drug resistance occur very frequently in these patients.

More recently, immunotherapies (immune checkpoint inhibitors or chimeric antigen receptor T cell therapies - CAR-T) were introduced as a novel therapeutic approach, and they are currently under investigation [76,77]. In particular, in the last two decades, several types of monoclonal antibodies (mAbs) against immune checkpoints were developed and approved by the FDA for the treatment of PCa. These include anti-CTLA-4 (cytotoxic T-lymphocyte-associated protein-4) (ipilimumab, tremelimumab) and anti-PD-1 (programmed death protein-1) (nivolumab, pembrolizumab, lambrolizumab, avelumab, durvalumab) mAbs. Clinical trials have been already performed in PCa patients treated with checkpoint inhibitors, either alone or in combination with antiandrogens [77,78,79,80,81,82,83,84,85,86,87,88]. Unfortunately, despite the initial success in clinical use of these compounds, the efficacy of treatments was reported to be low and the majority of PCa patients showed resistance to these therapies [87,89,90,91].

Studies leading to a better understanding of the molecular mechanisms and signaling pathways involved in PCa development and progression are needed to improve the chemopreventive/treatment strategies for this pathology.

## 3. GnRH Receptors in Prostate Cancer

### 3.1. Molecular Structure

The expression of the GnRH-R in human pituitary, as well as its nucleic acid sequence, was first reported by Kakar and coworkers in 1992 [92]. In the same years, it was becoming increasingly clear that the GnRH-R was expressed not only at the pituitary level but also in extrapituitary sites and in cancer tissues, including prostate cancer [34,37,39,44,93,94]. These receptors were first analyzed in terms of binding affinity for GnRH synthetic analogs, leading to contradictory results. In our laboratory, we demonstrated that one single class of low-affinity GnRH binding sites was present in PCa cells, either androgen-dependent or castration-resistant [95,96]. On the other hand, two classes of GnRH binding sites (one low affinity and one high affinity) were demonstrated in human PCa cells as well as in the Dunning R3327 rat model of PCa [97,98], while a single class of high affinity binding sites was observed in Dunning R3327 rats by Pinski and coworkers [99].

Based on these contrasting observations, further studies were performed to characterize this receptor at the molecular level. We could demonstrate the expression of a GnRH receptor, sharing the same mRNA and protein size with the pituitary receptor, in PCa cells [37,100,101,102]. Similar observations were later reported by Bank et al. in PCa cells [103], in the rat R3327 prostate adenocarcinoma and in human prostate biopsies [104,105,106,107,108]. Interestingly, a lower expression of GnRH-R was reported in normal prostate specimens when compared to PCa biopsies [109].

As reported for different types of tumors, the decapeptide GnRH is also expressed in PCa cells, further supporting the existence of a GnRH/GnRH-R autocrine loop involved in the local control of tumor growth and progression [96,100,101,110].

### 3.2. Antiproliferative/Proapoptotic Activity

The antitumor activity of GnRH-R in PCa cells is now well established. In our, as well as in others laboratories, activation of GnRH-R by means of GnRH agonists was shown to significantly inhibit the proliferation of human androgen-dependent (LNCaP) and CRPC (DU145, PC3) cells, expressing high levels of the GnRH-R, both in vitro [93,95,96,111,112] and in vivo, when subcutaneously inoculated in nude mice [113,114,115]. In line with these observations, GnRH analogs were also reported to inhibit the growth of the rat androgen-independent Dunning R-3327-AT-1 prostate cancer [99] as well as of primary cell cultures from human prostate carcinomas [116]. Moreover, GnRH agonist-based therapy was reported to be associated with longer survival in hormone-refractory PCa patients expressing the GnRH-R [111]. Interestingly, we demonstrated that the classical form of GnRH-R mediates the anticancer activity of CRPC cells, further supporting that a functional GnRH-II-R is not present in humans and that GnRH-II may act through the classical GnRH-R [117].

The antitumor effects of GnRH-R activation have been suggested to be associated not only with a slowdown of the cell cycle progression (mainly at the G2/M checkpoint) but also with induction of apoptosis. Specifically, GnRH agonists were shown to induce apoptosis in CRPC cells by interfering with the activity of both the PI3K pathway, leading to the stimulation of the downstream JNK kinase, and the p38 MAPK signaling cascade [118,119]. The extrinsic apoptotic pathway involving caspase 8 and 3 and p53 phosphorylation, was also reported to be induced by GnRH agonists in primary cultures of human PCas [116,119,120,121,122]. Interestingly, we could demonstrate that GnRH agonists sensitize, and resensitize, to chemotherapy (i.e., docetaxel) in a p53-dependent manner [123]. However, the involvement of the apoptotic pathways in the antitumor activity of GnRH-R in PCa is still a matter of debate [114,124].

Growth factors (EGF, IGF-I) and their receptors play a pivotal role in the growth and progression of tumors, including PCa [101,125,126,127,128,129,130,131]. To elucidate the molecular mechanisms underlying the anticancer effects of GnRH-R, the possible interference of GnRH analogs with the protumoral activity of growth factors has been investigated in different experimental models of PCa.

In our laboratory, it was demonstrated that activation of GnRH-R, in both androgen-dependent and CRPC cells in vitro and in vivo, interferes with the mitogenic activity of the locally expressed EGF stimulatory loop by decreasing the expression of EGF-R and of its downstream signaling molecules (i.e., the transcription factor *c*-fos) [113,114,128,132]. Similar observations were reported by Iacopino et al., showing that the GnRH agonist leuprolide significantly counteracts EGF-induced ERK1/2 phosphorylation (i.e., activation) in PCa cells [133]. The GnRH-R/EGF receptor interaction in PCa cells was further confirmed by Wells and coworkers [134]. The phosphorylation of the EGF-R at threonine 654 by the endogenous growth factor plays a central role in the receptor activation. To confirm the interference of GnRH-R activation with the EGF mitogenic activity, these authors overexpressed a mutant form of the EGF-R (threonine 654 to alanine) in DU145 CRPC cells. They demonstrated that the GnRH agonist goserelin counteracts the mitogenic activity of EGF in wild type DU145 cells (as expected), but not in cells overexpressing the mutant (i.e., inactive) form of the receptor [134].

The insulin-like growth factor (IGF) system is also known to be deeply involved in PCa growth and progression. This system is composed of two ligands (IGF-I and IGF-II), two receptors (IGFR-IR and IGFR-IIR) and different binding proteins (IGFBP-1 to -6). Mita and coworkers demonstrated that, in human PCa tissues, the expression of IGF-II and IGFBP-2 significantly correlates with pathologic stage lymph node metastasis, histologic differentiation and serum prostate-specific antigen (PSA) levels after hormone therapy [130]. On the other hand, a recent paper reported a significant correlation between IGF-IR expression in human PCa biopsies and tumor stage, suggesting that it may play a role in PCa progression towards an aggressive phenotype [135]. In our laboratory, we demonstrated that, in DU145 cells, activation of the GnRH-R with the agonist goserelin counteracts the mitogenic action of IGF-I in a dose-dependent manner, prevents the growth factor-induced tyrosine phosphorylation of the IGF-IR and decreases the expression of IGF-IR without affecting its binding affinity for the growth factor [37,136].

GnRH antagonists were first developed for the treatment of hormone-dependent PCa, based on their ability to compete with the binding of endogenous GnRH to its pituitary receptors, with the aim to suppress the activity of the pituitary gonadal axis without triggering the initial undesired gonadotropin surge. It was then expected that these compounds might also suppress the activity of the GnRH-R expressed in tumor tissues. Surprisingly, it was found that, in cancer cells expressing the receptor, these compounds exert a significant antiproliferative activity, supporting that they act as agonists on these cells [36,38,39,45,59,137,138]. Specifically, activation of GnRH-R by GnRH antagonists was also reported to suppress the proliferation, and to trigger apoptosis, by interfering with the growth factor receptor intracellular pathways in PCa cells [37,39,41,42,43,93,99,116,139,140,141,142,143]. More recently, Cucchiara and coworkers demonstrated that the GnRH antagonist degarelix significantly reduces the proliferation of C4-2-MDVR (castration- and enzalutamide-resistant) PCa cells expressing the androgen receptor variant AR-V7. Interestingly, degarelix also decreases the expression of the androgen receptor as well as of androgen steroidogenesis pathways in tumor tissues from PCa cell xenografts grown in severe combined immunodeficient (SCID) mice [144].

The observation that, in tumors cells, GnRH-R can be activated not only by GnRH agonists (as expected) but also by GnRH antagonists, pointed out that these receptors might be characterized by specific structural properties according to the cell context in which they are expressed. In particular, Millar et al. proposed the ligand-induced selective signaling concept. This concept foresees that, in different tissues (i.e., pituitary vs. cancer cells), the GnRH-R may adopt different structural conformations associated with selective binding of GnRH analogs and specific intracellular signaling pathways [137,145].

As mentioned above, in addition to the classical GnRH, a structural isoform of the peptide (i.e., GnRH-II) has been discovered in most vertebrates. In particular, GnRH-II is known to be expressed in different human tissues, normal and malignant, including PCa [37,39,146]. This peptide was shown to exert a significant antiproliferative and proapoptotic effect on PCa cells, both androgen-dependent and castration-resistant [117,119,120,146]. It is now generally accepted that the classical form of the GnRH-R mediates the antitumor activity of this peptide, confirming the notion that a functional GnRH-II receptor is lacking in human tissues, although this issue still remains a matter of debate [45,120,147].

More recently, based on docking experiments, a novel ligand and activator of the GnRH-R, GV1001, structurally unrelated to the GnRH decapeptide, has been identified. This peptide is a 16-amino acid fragment of hTERT, the human telomerase reverse transcriptase catalytic subunit. GV1001 was reported to bind and activate GnRH-R, thus triggering an antiproliferative and proapoptotic effect in PCa cells, both in vitro and in vivo, when inoculated in nude mice [148].

Taken together, these observations strongly support that locally expressed GnRH-R mediate the antitumor activity of GnRH decapeptide isoforms, as well as of novel peptides endowed with anticancer effects, in PCa cells.

### 3.3. Antimetastatic Activity

Locally expressed GnRH-R are also involved in the control of the metastatic behavior of PCa cells. In our laboratory, we demonstrated that GnRH agonists significantly reduce the migratory behavior of CRPC cells towards the extracellular matrix protein vitronectin (by haptotactic assays) as well as their ability to invade a reconstituted basement membrane [149]. We also found that, in these cells, GnRH agonists interfere with the expression and activation (i.e., tyrosine-phosphorylation) of the IGF-IR; counteract the IGF-I-induced phosphorylation of AKT (a kinase known to be involved in the prometastatic activity of the growth factor); abrogate the migratory and invasive behavior triggered by IGF-I; interfere with the effects of the growth factor on actin cytoskeleton organization, expression and cellular localization of integrins (i.e., αvβ3), and cell morphology [149].

In line with these observations, GnRH-R activation was reported to block the invasive behavior of CRPC cells induced by the fibroblast growth factor (FGF) [111]. This antimetastatic activity was suggested to be mediated by the inhibition of the plasminogen activator system by decreasing the enzymatic activity and the secretion of uPA (urokinase-type plasminogen activator) while increasing the expression levels of the protein PAI-1 (plasminogen activator inhibitor type-1) [150]. Enomoto and coworkers showed that both GnRH-I and GnRH-II peptides induce actin cytoskeleton remodeling, and decrease cell migration, through the activation of the classical form of the GnRH receptor [151]. Moreover, the effects of a GnRH agonist (leuprorelin) were also investigated on the expression levels of molecules involved in migration, invasion and cell-cell adhesion in both androgen-dependent (LNCaP) and castration-resistant (PC3) PCa cells. It was found that, in LNCaP cells, the GnRH agonists upregulate the expression of E-cadherin, β- and γ-catenin. On the other hand, the expression of these molecules was not affected in PC3 cells [152].

Interestingly, in addition to their antimetastatic/antiinvasive activity, GnRH-R were shown to be also endowed with antiangiogenic properties. Actually, we could demonstrate that GnRH receptors are also expressed on human umbilical vein endothelial cells (HUVECs), and that GnRH agonists reduce their proliferation and ability to form capillary-like tubes when stimulated by vascular endothelial growth factor (VEGF). These findings suggest that activation of GnRH-R triggers an antiangiogenic effect by counteracting the proangiogenic activity of the growth factor directly at the level of endothelial cells [153].

A potent antimetastatic activity was also reported by treating PCa cells with GnRH antagonists, further supporting the notion that these compounds behave as agonists at the level of the GnRH-R expressed in cancer cells. Treatment of CRPC cells with the GnRH-R antagonist cetrorelix reduces the invasiveness of DU145 cells overexpressing the full-length EGF-R, while increasing the expression levels of cell-cell adhesion molecules such as E-cadherin, α- and β-catenin and p120 [154]. In CRPC cells, Dondi et al. reported that GnRH antagonists are endowed with antimetastatic activities that are similar to those exerted by agonists in CRPC cells [150]. The expression of proteins involved in the cell-cell adhesion and angiogenic processes was also found to be affected by the GnRH antagonist degarelix in BPH (benign prostatic hyperplasia)-1 cells [143].

### 3.4. Intracellular Signaling Pathways

GnRH analog activities at the pituitary level (activation of the pituitary-gonadal axis) significantly differ from those at the cancer cell level (inhibition of cancer cell growth and metastatic behavior) suggesting that, according to the cell context, GnRH-R can be coupled to different G proteins and, therefore, to specific intracellular signaling pathways and molecular transducers [14,15,16,20,37,42,44,45,137,140,155,156].

As discussed above, GnRH-R activation in pituitary gonadotrope cells stimulates gonadotropin synthesis and release through the Gαq/11/PLC/PKC/MAPK signaling pathway. On the other hand, in cancer cells, and specifically in PCa cells, the GnRH-R has been found to be mainly coupled to a Gαi protein. Activation of this G protein counteracts cAMP accumulation thus triggering antitumor effects [16,20,37,45,102,157,158].

In our laboratory, we demonstrated that in both androgen-dependent (LNCaP) and castration-resistant (DU145) PCa cell lines, pertussis toxin completely abrogates the antiproliferative action of GnRH agonists. These compounds substantially antagonize the pertussis toxin-catalyzed ADP-ribosylation of a Gαi protein. GnRH analogs significantly counteract the forskolin-induced increase of intracellular cAMP levels [102].

Through the activation of the Gαi/cAMP pathway, GnRH analogs were reported to trigger the activity of a phosphotyrosine phosphatase, thus resulting in a decreased phosphorylation of the EGF-R (i.e., inactivation) [159]. In line with this observation, we could show that GnRH agonists significantly counteract the EGF and IGF-I-induced tyrosine phosphorylation of the EGF-R and IGF-I-R respectively, thus interfering with the mitogenic activity of the growth factors in PCa cells [132,149,160].

The GnRH-R-linked Gαi/cAMP pathway was also reported to mediate the effects of GnRH analogs on the MAPK (p38MAPK, ERK, JNK) signaling cascades known to play a pivotal role in cell growth, proliferation and apoptosis [161,162]. Specifically, the p38MAPK pathway was found to mediate the proapoptotic activity of GnRH analogs in BPH-1 cells while the ERK kinase was shown to be activated by GnRH agonists in immortalized human prostate cells engineered to overexpress the GnRH-R. JNK activation was also demonstrated to be involved in the proapoptotic effects of GnRH analogs in CRPC cells [38,42,44,118,119,122,158]. The activation of JNK was suggested to be mediated by inhibition of mixed-lineage kinase 3 (MLK3), the upstream activator of JNK [44,118].

Finally, GnRH analogs were shown to interfere with the activity of the PI3K/AKT intracellular pathway to suppress the proliferative and metastatic features of CRPC [149].

In addition to the Gαi/cAMP pathway, the Gαq/11/PLC/PKC signaling cascade also seems to be involved in the antitumor activity of the GnRH-R in PCa cells [14,118]. Sviridonov and coworkers reported that the PKCα, PKCβII and PKCε kinases are activated by GnRH in PCa cells in a more prolonged way than in gonadotrope cells. This is followed by ERK1/2, p38MAPK and JNK activation (mediated by reduced AKT phosphorylation) and redistribution from the cytosol and Golgi to the plasma membrane. These authors suggest that both a more sustained vs. transient expression, and a different distribution of PKCs in PCa cells vs. gonadotropes, are responsible for the different biological effects elicited by GnRH in these cell types [20,163].

Thus, although the Gαi/cAMP pathway remains the main intracellular signaling cascade coupled to the GnRH-R, additional pathways (i.e., Gαq/11/PLC/PKC) are now accepted to be involved in the antitumor activity of this receptor in PCa cells.

**Figure 1:**
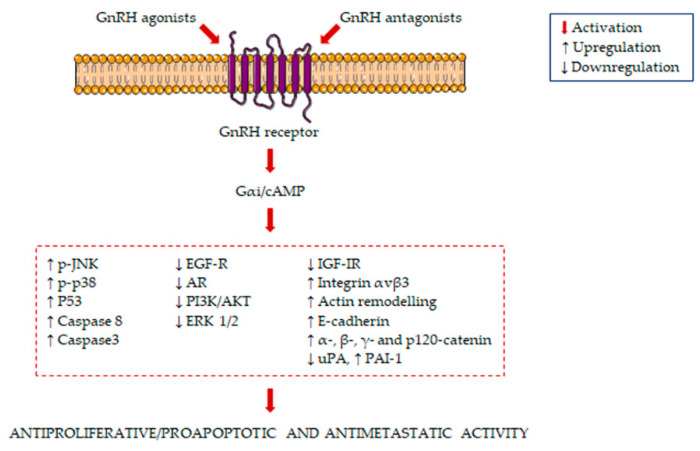
Molecular mechanisms underlying the antiproliferative/proapoptotic and antimetastatic activity of gonadotropin-releasing hormone receptors (GnRH) agonists and antagonists in prostate cancer (PCa) cells. GnRH agonists and antagonists bind to the GnRH receptor in PCa cells, leading to the activation of Gαi/cAMP. This is followed by the induction of several antiproliferative/proapoptotic and antimetastatic pathways.

The main signaling pathways activated in GnRH agonist/antagonist-treated PCa cells are summarized in Figure 1.

## 4. Emerging Prospective Aspects for New Therapeutic Interventions

### 4.1. GnRH Agonists and Antagonists

The expression of GnRH-R in PCa cells, specifically CRPC cells, together with their antitumor activity, sustained the hypothesis that they might represent an additional and direct molecular target of GnRH analogs (both agonists and antagonists) in CRPC [43]. Based on this notion, it has been proposed that discontinuation of ADT in CRPC patients could result in a worse outcome of tumor progression. Combination therapies based on ADT treatments together with chemotherapy, enzalutamide (antiandrogen) or abiraterone (inhibitor of androgen synthesis), are highly recommended [55]. Different clinical trials confirm this hypothesis.

It was reported that in CRPC patients receiving a GnRH agonist-based treatment, a high expression of GnRH-R was associated with a better disease-specific survival [111]. A retrospective study by Lawrentschuk et al. reviewed the records of PCa patients who were treated with a GnRH agonist (leuprorelin or goserelin) but underwent disease progression and were then rechallenged with the other GnRH agonist (goserelin or leuprorelin). These authors reported a significant decrease of PSA levels in patients undergoing this GnRH agonist-based switching therapy [164]. Interestingly, Teply and coworkers performed a clinical trial in which PCa patients progressing after an antiandrogen (enzalutamide) treatment were challenged with the bipolar androgen therapy (BAT, low testosterone levels together with a GnRH agonist). The results obtained showed positive clinical responses (as evaluated in terms of PSA levels and radiographic progression-free survival) and a subsequent resensitization to the antiandrogen [165]. In chemotherapy-naive patients with metastatic CRPC, the combined use of a GnRH agonist with docetaxel was shown to improve the median radiographic progression-free survival with respect to the chemotherapeutic drug given alone (nine vs. six months, respectively) [166]. In line with these data, patients who develop CRPC very often continue on a GnRH agonist-based androgen deprivation therapy when starting treatment with chemotherapy [167,168,169].

However, it should be underlined that contrasting results were reported by other clinical trials. For instance, in a large clinical trial (ICELAND), patients with locally advanced or relapsing PCa were treated with a GnRH agonist (leuprorelin), either alone or in combination with the antiandrogen bicalutamide. It was reported that in these patients, continuous androgen deprivation did not improve PSA progression [170].

As discussed in this review, GnRH antagonists bind to locally expressed GnRH-R in CRPC cells triggering the same antitumor effects elicited by GnRH agonists. Moreover, they induce a faster suppression of testosterone levels and are devoid of the undesirable initial testosterone surge. Interestingly, these compounds also induce a more significant and long-lasting reduction of FSH levels when compared with GnRH agonists and were suggested to interfere with the binding of the gonadotropin to its receptors in prostate cancer cells [171]. To this purpose, it must be underlined that both FSH and FSH receptors are expressed in PCa cells and tissues, suggesting their involvement in PCa development [172,173]. Moreover, hifh serum levels of FSU were reported to correlate with metabolic, cardiovascular, skeletal and cognitive effects [174]. Interestingly, in PCa patients, GnRH antagonists were recently reported to induce more significant suppressions of both FSH and PSA levels than those induced by GnRH agonists [175].

In a very recent paper, Abufaraj and coworkers reported that in PCa patients, GnRH antagonist treatments are associated with lower mortality rates and cardiovascular events as compared with GnRH agonists, while there are no differences in musculoskeletal events and fatigue. On the other hand, adverse reactions at the injection site are characteristic features of GnRH antagonists [176]. A lower toxicity of GnRH antagonists vs. agonists has been in additional articles [55,177,178,179,180].

Taken together, these observations paved the way for clinical studies addressing the anticancer potential of GnRH antagonists (i.e., degarelix, at present considered the most efficient antagonist for PCa due to its low histamine-releasing activity) in CRPC patients [181].

In a previous paper, the effects of degarelix were evaluated on PSA levels in patients experiencing a progression towards the CRPC stage after treatment with a GnRH agonist. Unfortunately, only a small number of cases was found to respond to this treatment [182]. On the other hand, a more recent paper reported that in a CRPC case, switching from a GnRH agonist to the antagonist degarelix is associated with a longer control of tumor progression [183]. Similar observations were reported by Atchia and coworkers in a systematic review and meta-analysis. By analyzing the data from thirteen clinical studies, these authors concluded that treatment with degarelix after failure of a GnRH agonist in patients progressing towards the CRPC stage, resulted in decreased or stable PSA levels in patients [184]. Thus, treatment with GnRH antagonists might be considered for patients with disease progression after GnRH agonist therapy. Interestingly, according to Uemura and coworkers, GnRH agonist/antagonist combination treatments represent the mainstay therapeutic approach in CRPC patients in Japan [185].

Clinical trials investigating the efficacy of GnRH analogs, either alone or in combination with standard strategies, in CRPC patients are at present ongoing (see https://www.clinicaltrials.gov/).

### 4.2. Cytotoxic GnRH-Based Bioconjugates

GnRH receptors expressed in cancer cells, and associated with antitumor activity, are now considered as an interesting molecular target for a new targeted therapeutic approach based on cytotoxic GnRH bioconjugates, compounds in which a GnRH derivative peptide is covalently linked to a cytotoxic drug. The rationale of this targeted therapeutic approach is that the GnRH peptide acts as a targeting moiety to specifically deliver the cytotoxic drug to cancer cells while sparing normal cells that do not express the GnRH receptor. Thus, GnRH binds to its receptor on tumor cell membranes, the bioconjugate is internalized by endocytosis and the cytotoxic drug is then released to enter the nucleus to exert its anticancer activity.

The bioconjugate AEZS-108 (also known as AN-152) consists of doxorubicin (Dox) coupled to the GnRH derivative via an ester bond. It was widely reported to exert a significant antitumor (antiproliferative, antimetastatic and antiangiogenic) activity in different types of cancer [60,186,187,188,189]. Specifically, this bioconjugate was shown to significantly decrease the proliferation and to induce apoptosis in both androgen-sensitive and CRPC cells in vitro and in vivo [187,190,191,192]. After internalization mediated by endocytosis, the ester bond of this compound is cleaved by carboxyilesterases releasing the free drug that, in turn, can accumulate in the nucleus to exert its cytotoxic activity [193]. In line with these observations, in phase I and II clinical trials, Liu and coworkers reported that AEZS-108, associated with acceptable safety properties, decreases PSA levels in CRPC patients facing disease progression after chemotherapy [194,195].

Similar results were obtained with the bioconjugate AN-207 consisting of 2-pyrrolino-doxorubicin coupled to [D-Lys^6^]-GnRH [187,188].

Novel GnRH-based bioconjugates were developed by coupling [D-Lys^6^]-GnRH to different anticancer compounds. Karampelas and coworkers developed a GnRH-based bioconjugate in which the decapeptide is linked to a molecule of gemcitabine, a drug with antitumor activity but with a fast metabolic inactivation. They reported that the conjugate exerts a significant antitumor activity in CRPC cells, in vitro and in vivo, associated with a relevant metabolic and pharmacokinetic advantage [196]. Argyros et al. analyzed the effects of a conjugate consisting of [D-Lys^6^]-GnRH and an analog of the antiangiogenic compound sunitinib (SAN1). These authors found that in mouse xenograft models of CRPC, this compound induces a significant delay in tumor progression when compared with equimolar sunitinib or SAN1 alone. Importantly, no cardiovascular side effects were observed during the treatment [197].

As stated above, the GnRH-III isoform found in sea lamprey is a decapeptide that differs from the classical form of GnRH in amino acids 5–8, with a lysine in position 8 (instead of arginine as in GnRH). GnRH-III binds to mammal GnRH-R at the pituitary level where it is endowed with a very poor gonadotropin releasing activity in mammals. On the other hand, it was shown to bind to GnRH-R expressed in cancer cells to trigger its antitumor effects [33,198].

Based on these observations, different anthracycline-GnRH-III bioconjugates were developed and characterized in terms of chemical and enzymatic stability, as well as in terms of their cytostatic effects. In first-generation bioconjugates, a molecule of daunorubicin (Dau) or Dox was linked to the ε-amino group present in ^6^Lys of the decapeptide by means of different chemical linkages (ester, oxime, amide bond). For instance, the oxime bond-linked Dau-GnRH-III bioconjugate was shown to possess a high chemical and enzymatic stability (in human serum as well as in the presence of rat liver lysosomal homogenates) as well as significant antitumor effects in several cancer cells, both in vitro and in vivo [199,200,201,202].

To increase the cytotoxic activity of these compounds, multifunctional anticancer drug-GnRH-III bioconjugates were developed with the aim to increase the stability and the antitumor effects of these compounds (i.e., chemical modifications of the targeting decapeptide and attachment of more than one cytotoxic drug). Leurs and coworkers reported the synthesis and development of a bifunctional [^4^Lys]-GnRH-III containing two lysine amino acids (in position 4 and 8) coupled with two Dau molecules through their ε-amino groups. The same authors also synthesized and characterized a bioconjugate in which the GnRH-III peptide was used as a scaffold and a second molecule of Lys was attached to the amino group of ^8^Lys to provide the binding sites for two Dau molecules [203,204]. Similarly, GnRH-III based multifunctional delivery systems obtained by linking two different anticancer drugs (i.e., Dau and metotrexate) or composed by Dau-GnRH-III derivative dimers were developed [205,206]. More recently, the development and the anticancer effects of different oxime bond-linked Dau-GnRH-III bioconjugates containing different unnatural amino acids were reported by Schuster and coworkers [207]. A high enzymatic stability, as well as a significant cytotoxic activity in different types of cancer cells, both in vitro and in vivo, was reported for all these compounds [203,204,205,206,207].

In our laboratory we investigated the anticancer activity of two GnRH-III bioconjugates on CRPC cells (Dau-GnRH-III, in which Dau is bound to ^8^Lys of GnRH-III; Dau-[^4^Lys(Ac)]-GnRH-III, in which ^4^Ser of the Dau-GnRH-III conjugate is replaced by an acethylated lysine). We demonstrated that, after a rapid internalization, both Dau-GnRH-III and Dau-[^4^Lys(Ac)]-GnRH-III exert a significant antiproliferative/proapoptotic activity, that is counteracted by the cotreatment with a GnRH-R antagonist or by silencing of the classical form of the GnRH-R [208].

Although further studies (both in vitro and in vivo) are needed to confirm their lack of toxicity as well as their anticancer effects, these observations support an important role of GnRH bioconjugates as a novel delivery approach of cytotoxic drugs in targeted cancer therapy.

**Figure 2:**
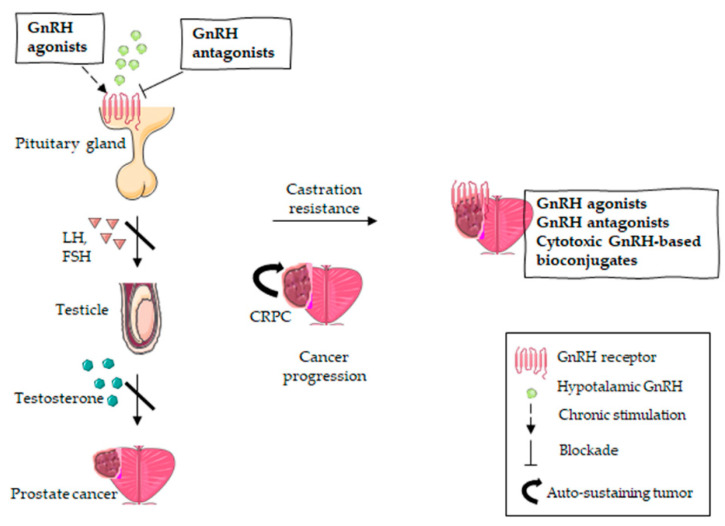
GnRH-R-targeted therapies in PCa treatment. Androgen-responsive PCa is targeted with GnRH antagonists (direct inhibition of the pituitary GnRH receptor) or GnRH agonists (chronic stimulation inducing receptor desensitization), resulting in the suppression of the pituitary axis. The inhibition of androgen production leads to tumor regression. However, PCa can become castration-resistant, which means tumor progression occurs despite the suppression of the pituitary axis. Given the expression of GnRH receptors on CRPC cells, the latter might be further specifically targeted with GnRH agonists, GnRH antagonists or cytotoxic GnRH-based bioconjugates.

Current GnRH-R-targeted strategies for the management of both androgen responsive PCa and CRPC are illustrated in Figure 2.

## 5. Conclusions

Pituitary GnRH-R mediate the pivotal role of the hypothalamic decapeptide GnRH in the control of the pituitary-gonadal axis functions. It has been widely shown that GnRH-R, sharing the same gene sequence, as well as mRNA and protein size, with the pituitary receptor, are also expressed in different tumors, such as PCa. Several data from the literature, from in vitro and in vivo studies, report that in PCa cells, and specifically in CRPC cells, GnRH agonists are associated with significant antiproliferative/proapoptotic, antimetastatic and antiangiogenic activities. Interestingly, GnRH antagonists were demonstrated to trigger the same antitumor effects. Millar and coworkers proposed that GnRH-R may adopt different structural conformations according to the cell context in which they are expressed, thus being associated with selective binding of GnRH analogs [137,145].

The opposite role of GnRH-R at the pituitary (stimulation of gonadotropin synthesis/secretion) and at the cancer level (antiproliferative effects) is related to the different intracellular signaling pathways associated with these receptors. In PCa cells these receptors are mainly associated with the Gαi/cAMP pathway, triggering the activity of a tyrosine phosphatase, and subsequent inactivation of tyrosine kinase receptors (i.e., EGF-R and IGF-I-R), and finally interfering with downstream different molecular pathways, such as the MAPK and PI3K/AKT signaling cascades.

Based on these molecular observations, GnRH agonists and antagonists, in combination with docetaxel, are currently considered as an effective therapeutic approach for chemotherapy-naive CRPC patients.

GnRH-based bioconjugates are now considered as a novel targeted therapeutic approach for CRPC. These are drugs in which a cytotoxic compound is covalently linked to a GnRH derivative peptide. It is believed that by binding to its receptors in cancer cells, the GnRH peptide can specifically deliver the chemotherapeutic drug to cancer cells while sparing normal cells not expressing the GnRH receptors. In this context, the most studied bioconjugate is AEZS-108 (AN-152), consisting of a molecule of doxorubicin linked to [D-Lys^6^]-GnRH. This bioconjugate was demonstrated to exert a significant antitumor activity in CRPC cells in vitro and in vivo. Results from phase I and II clinical studies support these experimental observations. Bioconjugates based on different isoforms of the GnRH peptide, such as GnRH-III, are also under investigation.

Further studies are needed to obtain novel compounds with a high specific efficacy and low toxicity to improve the treatment options for CRPC patients.

## Figures and Tables

**Table 1:** Human Gonadotropin-releasing hormone (GnRH) receptors.

Name of Gene	*GNRHR*	*GNRHR2*
Chromosome location	4q13.2	1q12
Name of protein	GnRH-R	GnRH-II-R
Length	328aa	292aa

GNRHR2 shares 41% sequence identity with GNRHR.
